# Association of E26 Transformation Specific Sequence 1 Variants with Rheumatoid Arthritis in Chinese Han Population

**DOI:** 10.1371/journal.pone.0134875

**Published:** 2015-08-04

**Authors:** Lin Chen, Zhuochun Huang, Bin Yang, Bei Cai, Zhenzhen Su, Lanlan Wang

**Affiliations:** Department of Laboratory Medicine, West China Hospital, Sichuan University, Chengdu, China; University of Birmingham, UNITED KINGDOM

## Abstract

**Objective:**

E26 transformation specific sequence 1 (ETS-1) belongs to the ETS family of transcription factors that regulate the expression of various immune-related genes. Increasing evidence indicates that ETS-1 could contribute to the pathogenesis of autoimmune disease. Recent research has provided evidence that ETS-1 might correlate with rheumatoid arthritis (RA), but it's not clearly defined. In this study, we aimed to identify whether polymorphisms of ETS-1 play a role in Rheumatoid arthritis (RA) susceptibility and development in Chinese Han population.

**Methods:**

Four single nucleotide polymorphisms (SNPs) within ETS-1 were selected based on HapMap data and previous associated studies. Whole blood and serum samples were obtained from 158 patients with RA and 192 healthy subjects. Genotyping was performed with polymerase chain reaction-high resolution melting (PCR-HRM) assay and the data was analyzed using SPSS17.0.

**Results:**

A significantly positive correlation was observed between the SNP rs73013527 of ETS-1 and RA susceptibility, DAS28 and CRP (P<0.001, P = 0.001, and P = 0.028, respectively). Carriers of the haplotype CCT or TCT for rs4937333, rs11221332 and rs73013527 were associated with decreased risk of RA as compared to controls. No statistical significant difference was observed in the distribution of rs10893872, rs4937333 and rs11221332 genotypes between RA patients and controls.

**Conclusions:**

Our data further supports that ETS-1 has a relevant role in the pathogenesis and development of RA. Allele T of rs73013527 plays a protective role in occurrence of RA but a risk factor in the high disease activity. Rs10893872, rs11221332 and rs4937333 are not associated with RA susceptibility and clinical features.

## Introduction

Rheumatoid arthritis (RA) is a complicated autoimmune disease, whose clinical course ranges from mild joint swelling to severe poly-arthritis with progressive destruction of cartilage and bone. It is a serious pathological condition that can lead to incapacitation and decreased life expectancy compared with the normal population [1]. People suffering from RA weigh around 1% of world’s population [2]. In China, the prevalence of RA in the overall population is 0.32%-0.36%. What’s more, according to the data published by Ministry of Health of China, RA is one of the major chronic diseases in rural area of China. So far, the etiology of RA has yet to be clarified, but it has been established that genetic factors play a role in susceptibility to this condition [3], and the genetic variants may account for 50%–60% of the etiology [4].

To date, many genes have been identified to be associated with RA and the main genetic factor is the HLA-DRB1 gene, which accounts for one-third of the genetic liability to the disease [5]. Besides HLA-DRB1, non-HLA genes (e.g. PTPN22, NFAIP3, STAT4, TRAF1/C5, IL2RB, AFF3, CD40, CTLA4, MMEL1 and PADI4) have also been implicated in disease susceptibility, but many other genes still remain to be discovered [6]. Heo SH and their colleagues had confirmed that E26 transformation specific sequence 1 (ETS-1) was involved in the regulation of matrix metalloproteinases (MMPs) [7] which played a direct and important role in tissue destruction[8]. Recently, studies suggested that ETS-1 might be a crucial factor in the cytokine-mediated inflammatory and destructive cascade characteristic of RA [9, 10].

ETS-1 belongs to the ETS family of transcription factors. It mainly expresses in lymphoid cells and plays multiple roles in the lymphocyte development, function, apoptosis and inflammation [11–14]. Many viral and cellular genes, including those encoding growth factor genes and proto-oncogenes, are activated by ETS-1. At the same time, the regulation genes of some important immune functional proteins are activated (TCRα, Cd3δ, IL-5, Lck, c-fms, mb1, Igh, csf2) or repressed (TCRβ, IL-2) by ETS-1 as well [15, 16]. Recently, studies demonstrated that ETS-1 is a factor in causing the SLE in East Asian [17, 18]. Meanwhile, some single nucleotide polymorphisms (SNPs) of ETS-1 were identified as the susceptible variants of some immune diseases. For example, rs10893872, rs4937333 of ETS-1 were found as significant variants associated with SLE susceptibility [17, 19]. Rs10893872 may affect the genetic predisposition to pediatric uveitis [20] and rs11221332 is related with celiac disease and RA in Caucasians [10, 21]. Also, recent Genome-Wide Association studies (GWAS) revealed that rs73013527 associated with RA in Europeans [22]. All these diseases mentioned above are characterized by excessive activation of the immune system.

Therefore, ETS-1 is a very promising research field for understanding diseases of immune deficiency and autoimmunity. The aim of the present study is to identify the role of the ETS1 polymorphisms rs73013527, rs10893872, rs4937333 and rs11221332 in RA in Chinese Han population.

## Materials and Methods

### Participants

Our study recruited 350 Chinese Han subjects, including 158 RA patients and 192 healthy controls, from September 2012 to September 2014 in West China Hospital. All the subjects to be enrolled in the case-control study signed informed consents. All patients fulfilled rheumatoid arthritis criteria 2010 released by ACR/EULAR [23]. Controls were randomly selected by inviting people who lived in the same area as the patients to take part in the study. Individuals were considered healthy if their medical history did not reveal any chronic diseases, endemic infectious diseases or autoimmune diseases and their physical examination and blood tests prompt normal. Otherwise, any individual with one or more of these conditions was excluded from the control group. The study was approved by the Ethics Committee of West China Hospital.

### Patient information

The following information was gathered from the patients’ medical records and collated on a form specifically prepared for the purpose: age, gender, disease duration (DD), age at onset (AO), painful joint and swollen joint. Moreover, many tests were done to get more information including rheumatoid factor (RF), anti-cyclic citrullinated peptide antibody (anti-CCP), erythrocyte sedimentation rate (ESR) and C-reactive protein (CRP) during assessment. Disease activity was determined according to the disease activity score for 28 painful/swollen joints (DAS28) [24].

### ETS-1 polymorphisms genotyping

All the four SNPs (rs10893872, rs4937333, rs11221332, rs73013527) were genotyped using polymerase chain reaction-high resolution melting (PCR-HRM) and analysis performed on Light Cycler 480 (Roche Diagnostics, Penzberg, Bavaria, Germany). Genomic DNA kit (Biotake Corporation, Beijing, China) was used to extract the free circulating DNA from the blood sample and the concentration was measured by Nanodrop 2000c spectrophotometer (Thermo Scientific, DE). SNP genotyping was performed in a 20μL reaction system contains 10μL Roche Master Mix (Roche Applied Science) which comprises FastStart Taq DNA Polymerase and the High Resolution Melting Dye in a reaction buffer, 2.4μL 25 mM MgCl2, 0.2μL 10 mmol/L Forward Primer and 0.2μL 10 mmol/L Reverse Primer, 6.2μL deionized water and finally 1μL DNA sample as recommended by the manufacturer. The whole genotyping process encompasses four programmes, namely, pre-denaturation, amplification, high resolution melting and cooling. It was performed under the following conditions: an initial denaturation step at 95°C for 10 min, then continued with 50 cycles of 95°C for 15 s, touchdown cycling (decreasing 1°C/cycle), annealing in the range of 65–55°C for 10 s, and 72°C for 10 s. After the amplification phase, PCR products were denatured at 95°C for 1 min and cooled to 40°C for 1 min to form double-stranded DNA. Then the HRM analyses were performed by gradually increasing the temperature from 65 to 95°C at a rate of 0.01°C/s. After the melting process, the instrument was cooled down to 40°C. When finished, the results were analyzed by the corresponding Gene Scanning Software v1.2 (Roche Diagnostic) primarily based on the shape of the melting curve.

### Laboratory assays

Serology markers of RA were analyzed using the following methods: RF and CRP were tested using Beckman Coulter IMMAGE 800 immunoassay (Beckman Coulter,Inc, CA, USA). Anti-CCP was analyzed by Elecsys Modular E170 immunoassay (Roche Diagnostics, GmbH, Mannheim, Germany). Anti-keratin antibody (AKA) was tested by indirect immunofluorescence. All the tests were conducted in accordance with manufacturers’ instruction.

### Statistical analysis

Hardy-Weinberg equilibrium was independently appraised for each polymorphism. Demographic and clinical data between groups were compared by Student’s t test or Mann-Whitney U test for continuous variables, as appropriate. Haploview software was used to explore whether ETS-1 polymorphisms were in strong linkage disequilibrium (LD) or they independently contribute to the susceptibility of RA, and can capture additional significant variants since it’s more sensitive than the single SNP analysis. Pearson’s chi-square test or Fisher’s exact test were used to analyze the allele case-control comparisons. Association of SNPs with development as well as susceptibility of RA was estimated by figuring out the odds ratio (OR) and 95% Confidence Interval (CI). When comparing the two groups of subjects (case and control), several analytic methods were used: allele frequency distribution of the two groups (allele A versus allele B, A as the major allele, B as the minor allele, this applied to the following methods); dominant model (AB+BB versus AA); recessive model (AA+AB versus BB). All statistical analyses were performed using the Statistical Package for the Social Sciences (SPSS, SPSS Inc., Chicago, IL, USA), version 17.0. A two-sided P value <0.05 was deemed as statistically significant.

## Results

### Characteristics of the study population

A total of 158 RA patients and 192 healthy controls were included in the study. Their main demographic and clinical characteristics were showen in Table 1 and S1 Table. Subjects were adequately matched for age and sex between RA patients and controls (P = 0.953 and P = 0.731, respectively). The mean age in RA patients and controls were 54.4 and 55.1 years. The age at onset of case group was 43.9±14.4 years and disease duration is 10.5±9.9 years. The mean of erythrocyte sedimentation rate for patients is 70.5 mm/h (SD±34.6) and mean of DAS28 score is 6.32 (SD±1.62). Furthermore, 84.81% of Patients were rheumatoid factor (RF) positive and 83.54% were anti-cyclic citrullinated peptide (anti-CCP) positive. The positive percentage of c-reactive protein (CRP) and anti-keratin antibody (AKA) in RA patients is 81.01% and 45.95%, respectively.

**Table 1:** The main demographic and clinical characteristics of patients and controls.

Characteristics	Case	Control	P value
**Age, mean±SD (years)**	54.4±12.3	55.1±9.8	0.953
**Female(%)/Male(%)**	76.6/23.4	75.0/25.0	0.731
**Age at onset, mean±SD (years)**	43.9±14.4	-	-
**Disease duration, mean±SD (years)**	10.5±9.9	-	-
**DAS28, mean±SD**	6.32±1.62	-	-
**RF-positive (%)**	84.81	-	-
**AntiCCP-positive (%)**	83.54	-	-
**AKA-positive (%)**	45.95	-	-
**CRP,mean±SD (%)**	34.8±43.5	-	-
**ESR, mean±SD (mm/h)**	70.5±34.6	-	-

Note: a) Data were expressed as median ± SD or median (interquartile range).

b)-: data were not available.

Abbreviations: DAS28, disease activity score for 28 painful/swollen joints; RF, rheumatoid factor; AntiCCP, anti-cyclic citrullinated peptide; CRP, c-reactive protein; AKA, anti-keratin antibody; ESR, erythrocyte sedimentation rate.

### Genotyping and LD evaluation

In this study, all subjects were clearly genotyped using PCR-HRM methods for the four SNPs and the correctness of assays was verified by direct sequencing for PCR products of randomly selected samples. Sequencing results were in complete accord with all the corresponding genotypes. No significant deviation was found from Hardy-Weinberg equilibrium (HWE), as determined at the 0.05 significance level.

**Fig 1:**
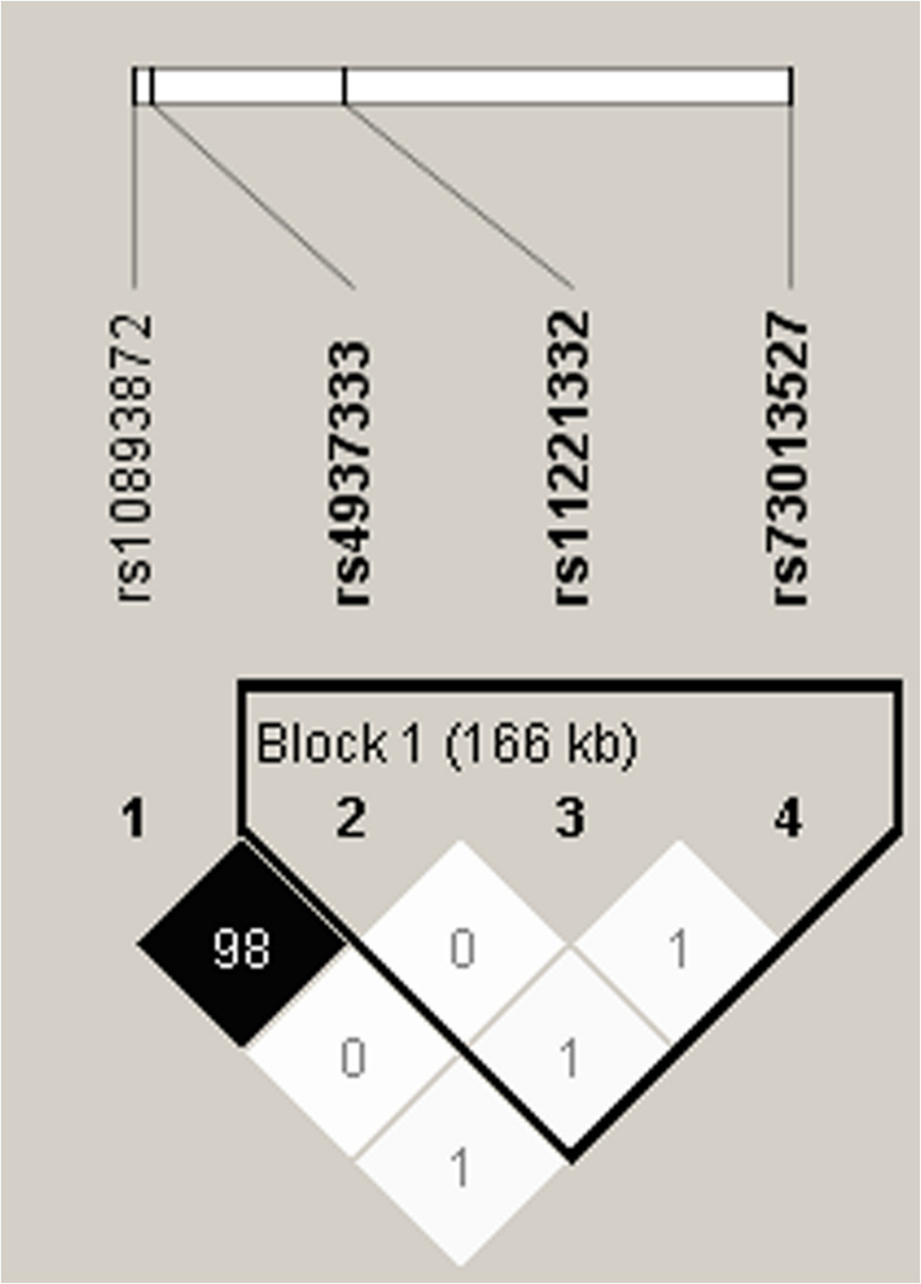
Linkage disequilibrium for four SNPs in 350 individuals. The linkage disequilibrium plot shows r² values between each pair of SNPs. rs10893872 and rs4937333 were in strong LD (black squares). D’ = 1.000, r2 = 0.980.

Using Haploview to conduct linkage disequilibrium evaluation, two SNPs (rs10893872 and rs4937333) in ETS-1 were in complete linkage disequilibrium (Fig 1). Therefore, the analyses were only carried out on the rs4937333 for its higher minor allele frequency (MAF = 0.44 and 0.42 for rs4937333 and rs 10893872, respectively).

### Association between ETS-1 SNPs and the risk of RA

Genotype distributions of rs73013527 were found to be statistically different between RA patients and the controls. The frequencies of the two groups differed significantly (dominant model, OR = 0.43, 95%CI = 0.28–0.66, P<0.001; recessive model, OR = 0.44, 95%CI = 0.23–0.84, P = 0.012; allelic model, OR = 0.51, 95%CI = 0.37–0.70, P<0.001) (Table 2).

However, none of rs4937333 and rs11221332 polymorphisms achieved a significant difference in the genotype distributions between cases and controls (dominant model, OR = 0.92, 95%CI = 0.59–1.45, P = 0.725 and OR = 0.82, 95%CI = 0.43–1.56, P = 0.546, respectively) (Table 2).

**Table 2:** Genotype distributions of ETS-1 in RA patients and Controls in Chinese Han population.

SNPs	Model	Genotype	RA (n = 158)		Controls(n = 192)		OR(95%CI)	P value
			N	%	N	%		
rs73013527	Dominant	CC	87	55.1	66	34.4	1.00	
		CT+TT	71	44.9	126	65.6	0.43(0.28–0.66)	**<0.001**
	Recessive	TT	14	8.9	35	18.2	1.00	
		CC+CT	144	91.1	157	81.8	0.44(0.23–0.84)	**0.012**
	Allele	C	231	73.1	223	58.1	1.00	
		T	85	26.9	161	41.9	0.51(0.37–0.70)	**<0.001**
rs4937333	Dominant	CC	53	33.5	61	31.8	1.00	
		CT+TT	105	66.5	131	68.2	0.92(0.59–1.45)	0.725
	Recessive	TT	19	12.0	30	15.6	1.00	
		CC+CT	139	88.0	162	84.4	0.74(0.40–1.37)	0.334
	Allele	C	192	60.8	223	58.1	1.00	
		T	124	39.2	161	41.9	0.90(0.66–1.21)	0.472
rs11221332	Dominant	CC	140	88.6	166	86.5	1.00	
		CT+TT	18	11.4	26	13.5	0.82(0.43–1.56)	0.546
	Recessive	TT	0	0.0	2	1.0	-	
		CC+CT	158	100.0	190	99.0	-	0.503
	Allele	C	298	94.3	356	92.7	1.00	
		T	18	5.7	28	7.3	0.77(0.42–1.42)	0.397

Abbreviation: OR, odds ratio; CI, confidence interval.

Note: a) Data were presented as number and percentage for every group.

b) Significant p-values (<0.05) are highlighted in bold.

### Haplotype analysis of ETS-1 with RA susceptibility

Haplotypes were constructed on the basis of 3 SNPs (rs4937333, rs11221332 and rs73013527) and four common haplotypes were inferred out (frequency>5%). The most common haplotype was CCC, whose frequency was 0.342. Meanwhile, the other three haplotypes were TCC, CCT and TCT according to the frequency order (Table 3).

Moreover, the results of haplotype analysis showed that haplotype block CCT and TCT significantly correlated with reduced risk of RA susceptibility (CCT: OR = 0.505, 95%CI = 0.329–0.775, P = 0.002; TCT: OR = 0.356, 95%CI = 0.206–0.615, P<0.001) (Table 3), which was in line with the results of genotype analyses.

**Table 3:** Haplotype analysis of 3 ETS-1 polymorphisms in Chinese Han population.

Haplotype*	All(freq)**	Case(freq)	Control(freq)	OR(95%CI)	P value
CCC	239.3(0.342)	127.3(0.403)	112.0(0.292)	1.000	
TCC	195.2(0.279)	96.6(0.306)	98.6(0.257)	0.864(0.592–1.261)	0.449
CCT	139.8(0.200)	51.2(0.162)	88.6(0.231)	0.505(0.329–0.775)	0.002
TCT	79.7(0.114)	22.9(0.072)	56.8(0.148)	0.356(0.206–0.615)	<0.001

Abbreviation: OR, odds ratio; CI, confidence interval.

Note: a) Data were presented as number (percentage) for every group. A two-sided p value < 0.05 was considered as statistically significant.

b) * All frequencies <0.03 had been ignored in analysis. Loci chosen for hap-analysis were in this order: rs4937333, rs11221332, rs73013527.

c) **Data exhibited haplotype frequencies (percentage) in the whole 350 subjects.

### Association of ETS-1 polymorphisms with clinical characteristics in RA patients

Table 4 summarizes the influence analysis of ETS-1 polymorphisms on clinical features of RA patients which shows that rs73013527 was associated with DAS28 and CRP (P = 0.001 and P = 0.028, respectively). These two indexes of different genotype group of rs73013527 differed significantly (DAS28: recessive model, P<0.001; CRP: dominant model, P = 0.022; recessive model, P = 0.034). However, for RF and anti-CCP, not so conspicuous difference as DAS28/CRP was observed between the genotypes of rs73013527 (RF: P = 0.821; anti-CCP: P = 0.351). No significant association was detected between rs4937333/rs11221332 and DAS28, RF, CRP and anti-CCP (DAS28: P = 0.127 and P = 0.423; RF: P = 0.313 and P = 0.874; CRP: P = 0.983 and P = 0.860; anti-CCP: P = 0.777 and P = 0.262, respectively). (Table 4).

**Table 4:** Association of ETS-1 polymorphisms with clinical characteristics in RA patients.

SNPs	Model	Genotype	DAS28		RF(IU/L)		CRP(mg/L)		Anti-CCP(U/L)	
			Md±IQR	P Value	Md±IQR	P Value	Md±IQR	P Value	Md±IQR	P Value
rs73013527		CC	6.50±1.53		344.00±1527.45		11.05±38.67		307.15±398.05	
		CT	6.62±2.21	**0.001**	323.00±1123.8	0.821	18.70±55.53	**0.028**	371.40±257.45	0.351
		TT	7.81±1.01		465.00±939.5		49.5±42.68		369.80±235.82	
	Dominant	CC	6.50±1.53		344.00±1527.45		11.05±38.67		307.15±398.05	
		CT+TT	6.80±1.84	0.162	366.00±1077.80	0.952	21.60±57.72	**0.022**	371.40±249.90	0.15
	Recessive	TT	7.81±1.01		465.00±939.5		49.5±42.68		369.80±235.82	
		CC+CT	6.54±1.62	**<0.001**	342.00±1140.45	0.572	15.60±41.99	**0.034**	351.10±382.70	0.509
rs4937333		CC	6.67±1.69		342.00±1535.25		16.10±49.36		328.60±403.54	
		CT	6.52±1.98	0.127	310.00±923.55	0.313	18.50±44.75	0.983	363.40±376.00	0.777
		TT	6.71±1.66		635.00±1407.5		10.7±55.9		350.10±220.5	
	Dominant	CC	6.67±1.69		342.00±1535.25		16.10±49.36		328.60±403.54	
		CT+TT	6.58±1.65	0.354	410.00±986.62	0.592	17.05±44.21	0.859	361.65±327.32	0.585
	Recessive	TT	6.71±1.66		635.00±1407.5		10.7±55.9		350.10±220.5	
		CC+CT	6.56±1.70	0.134	329.00±1129.12	0.216	17.65±46.32	0.926	361.65±383.3	0.559
rs11221332		CC	6.60±1.65		342.00±1188.85		16.7±47.32		340.10±379.7	
		CT	6.73±1.37	0.423	431.00±1015.2	0.874	17.20±48.73	0.860	416.65±242.5	0.262
	Dominant	CC	6.60±1.65		342.00±1188.85		16.7±47.32		340.10±379.7	
		CT+TT	6.73±1.37	0.423	431.00±1015.2	0.874	17.20±48.73	0.860	416.65±242.5	0.262

Abbreviation: Md±IQR: median±quartile interval.

Note: Significant p-values (<0.05) are highlighted in bold.

## Discussion

RA is a complex heterogeneous chronic autoimmune disease, whereby both environmental and genetic factors contribute to the etiology and/or clinical severity. The exact immunopathogenesis of RA has been one of the hottest research topics all the time. In this study, we have demonstrated that ETS-1 is associated with RA in Chinese Han population.

We identified a SNP, rs73013527 of ETS-1, which was significantly associated with RA in Chinese Han population, with minor allele T being correlated with a reduced risk of RA susceptibility. To our knowledge, rs73013527 wasn’t found to be correlated with other autoimmune disease and this is the first report demonstrating an association of this SNP of ETS-1 and RA in Han Chinese. In previous reports, some SNPs of ETS-1 were found to be significantly with many autoimmune diseases such as SLE, pediatric uveitis and celiac disease. In agreement with these autoimmune diseases, we observed that ETS-1 was also a susceptibility gene for RA at least in southern Han Chinese. This finding provides a support for that ETS-1 is a susceptibility gene for many autoimmune diseases. As we know, ETS-1 is a transcription factor and a member of the ETS family that activate transcription by binding to cis-regulatory elements in target genes [25]. It was initially discovered as the proto-oncogene corresponding to v-ets of the avian erythroblastosis virus (E26), which contains a conserved DNA-binding domain mediating specific DNA binding to the GGAA/T motif [26, 27]. Concerning the important roles of ETS-1, some researches indicated it might play a crucial role in RA. For example, ETS-1 was reported to be overexpressed in RA synovial membrane and to be involved in the destructive pathway of RA [28], but it was not clearly defined. In this study, the decreased frequency of the rs73013527 TT genotype in patients suggests that ETS-1 may be a predisposing factor in RA. What’s more, recent research has demonstrated that SNPs at miRNA binding sites likely affect the expression of the miRNA target genes and thus, may contribute to susceptibility to autoimmune diseases [29]. Since rs73013527 is located upstream of the ETS-1 gene and would not affect the sequence of the ETS-1 mRNA and hence could not affect miRNA binding. Therefore, we presumed that the SNP might influence the activity of an upstream enhancer of ETS-1 or might be in linkage disequilibrium with another genetic alteration that influences expression or activity of ETS-1. However, the detailed mechanism of this functional relationship requires further investigation.

Meanwhile, a significant correlation was found between rs73013527 and DAS28 as well as CRP, with minor allele T associated with an increase risk of disease activity. The TT genotype might influence the development of RA by a lasting high disease activity. It is interesting that minor allele T of rs73013527 plays a protective role in occurrence of RA but as a risk factor of high disease activity. A similar phenomenon was found in other researches. For example, Systemic lupus erythematosus (SLE) is a sexually dimorphic autoimmune disease which is more common in women, but male patients often experience a more severe disease state. Amr Sawalha speculated that men require a higher cumulative genetic load than women to develop the disease [30, 31]. The dimorphism of rs73013527 in RA is not clearly defined and it may correlate with gene-gene interaction and complex immune microenviroment, which worth to be studied further.

Although other studies showed that ETS-1 SNP rs11221332 has a positive association with RA susceptibility in Caucasians [10], it seemed not to be correlated with RA susceptibility in Chinese Han population in our study. It may be explained by that the risk variants are often population-specific and the difference can be truly originate from ethnic disparity since the degree of genetic variations differs among individuals of different ethnicities [32]. Besides, no significant correlation was found in the genotype, allele or haplotype frequencies of rs4937333 between RA patients and controls in our study. Considering that rs4937333 was significantly associated with SLE in East Asian populations, there were probably different target genes and pathway of regulation in these two similar diseases. Moreover, our study didn’t find the association between rs11221332 or rs4937333 and the clinical features such as DAS28, CRP, RF and anti-CCP.

As a crucial transcription factor, ETS-1 widely expressed in lymphocytes, vascular endothelial and various invasion tumor cells. It regulates the development, senescence and death of many immune cells, and also plays a role in both innate and adaptive immune response [25, 33]. Accumulating evidence points to an important role for ETS1 in regulating the differentiation of immune cells such as T-cell differentiation into a helper population, terminal differentiation of B cells, development of natural killer (NK) cells and NK T cells and the expression of cytokine and chemokine genes in a wide variety of different cell lineages [16, 25, 34–38]. What’s more, animal experiments showed that autoimmune disease developed in ETS-1 knockout mice, as investigated by the production of high titers of autoantibodies, and immune cell infiltration into organs accounted for aberrations in lymphocyte differentiation [36]. Since RA is a disorder of T cell dysfunction and systemic inflammation, ETS-1 might play a role in occurrence and development of RA. We will investigate this hypothesis in next study.

In summary, our study identified that the common variant (rs73013527) of ETS-1 confer susceptibility to RA. This SNP of ETS-1 also associated with the development of RA. This study might provide further evidence to improve our understanding of the exact function of ETS-1 in the pathogenesis of autoimmune diseases. It is worthwhile to mention that there are several limitations in our present study. The sample of patients in our study is relatively small and only Han Chinese cohorts in west China are included. Therefore, further studies in a large sample size and other ethnic populations are needed to confirm the results observed in this study.

## Supporting Information

S1 Table The demographic and other clinical characteristics of RA patients. (DOC)
