# Supplementary material for: Association of E26 Transformation Specific Sequence 1 Variants with Rheumatoid Arthritis in Chinese Han Population
Source: PLoS One. 2015 Aug 4;10(8):e0134875. doi: 10.1371/journal.pone.0134875 (PMC4524679; doi:10.1371/journal.pone.0134875)
Supplement: S1 Table — (DOC) [file pone.0134875.s001.doc]

**S1 Table. The demographic and other clinical characteristics of RA patients.**

| **NO.** | **Sex** | **Age** | **the number of painful joints** | **the number of swollen joints** | **TG(mmol/L)** | **TC(mmol/L)** | **HDL-C(mmol/L)** | **LDL-C(mmol/L)** |
| --- | --- | --- | --- | --- | --- | --- | --- | --- |
| 01 | F | 63 | 20 | 20 | 0.94 | 3.04 | 1.05 | 1.49 |
| 02 | F | 72 | 14 | 14 | 1.69 | 6.28 | 1.64 | 4.32 |
| 03 | F | 49 | 26 | 26 | 2.44 | 6.40 | 1.28 | 4.67 |
| 04 | F | 70 | 14 | 2 | 0.94 | 4.30 | 1.05 | 2.95 |
| 05 | F | 63 | 20 | 8 | 0.91 | 3.79 | 1.11 | 2.37 |
| 06 | M | 68 | 10 | 10 | 1.84 | 4.34 | 1.17 | 2.61 |
| 07 | F | 42 | 18 | 10 | 2.30 | 4.31 | 1.58 | 2.01 |
| 08 | F | 47 | 10 | 10 | 0.59 | 2.82 | 0.86 | 1.80 |
| 09 | M | 47 | 15 | 9 | 1.32 | 4.37 | 1.17 | 2.77 |
| 10 | F | 61 | 7 | 5 | 1.60 | 4.49 | 1.16 | 2.61 |
| 11 | F | 67 | 16 | 10 | 0.72 | 4.37 | 1.90 | 2.11 |
| 12 | F | 58 | 10 | 10 | 1.67 | 4.69 | 1.18 | 3.04 |
| 13 | M | 66 | 22 | 22 | 1.71 | 5.47 | 1.59 | 3.36 |
| 14 | M | 51 | 6 | 6 | 1.08 | 3.31 | 1.14 | 1.88 |
| 15 | F | 59 | 18 | 0 | 0.95 | 3.64 | 1.26 | 1.99 |
| 16 | F | 56 | 16 | 2 | 1.15 | 4.49 | 0.93 | 3.17 |
| 17 | F | 51 | 12 | 10 | 1.61 | 6.35 | 1.66 | 4.51 |
| 18 | M | 58 | 23 | 22 | 0.63 | 4.16 | 1.40 | 2.52 |
| 19 | F | 47 | 23 | 22 | 0.78 | 3.70 | 0.69 | 1.45 |
| 20 | F | 68 | 16 | 1 | 1.39 | 3.43 | 0.80 | 2.04 |
| 21 | F | 42 | 24 | 0 | 1.86 | 4.04 | 0.75 | 2.61 |
| 22 | F | 35 | 21 | 21 | 0.92 | 3.50 | 1.02 | 2.09 |
| 23 | M | 67 | 18 | 16 | 0.78 | 4.12 | 1.26 | 2.59 |
| 24 | F | 49 | 18 | 18 | 1.02 | 3.66 | 1.52 | 1.85 |
| 25 | F | 52 | 14 | 14 | 0.61 | 3.32 | 1.36 | 1.96 |
| 26 | M | 40 | 16 | 0 | 0.97 | 4.76 | 0.59 | 3.28 |
| 27 | F | 56 | 28 | 10 | 1.19 | 4.77 | 1.33 | 2.98 |
| 28 | F | 50 | 7 | 7 | 5.32 | 4.66 | 0.83 | 1.95 |
| 29 | F | 59 | 12 | 6 | 1.19 | 5.17 | 2.08 | 2.86 |
| 30 | F | 66 | 15 | 5 | 1.46 | 4.59 | 1.48 | 2.56 |
| 31 | F | 69 | 10 | 0 | 0.62 | 3.27 | 2.09 | 1.16 |
| 32 | F | 60 | 1 | 1 | 0.85 | 3.18 | 1.32 | 1.48 |
| 33 | F | 57 | 16 | 0 | 0.80 | 4.27 | 2.07 | 2.06 |
| 34 | F | 22 | 0 | 0 | 0.85 | 2.85 | 1.38 | 1.63 |
| 35 | F | 59 | 23 | 0 | 0.88 | 4.32 | 1.53 | 2.40 |
| 36 | M | 64 | 11 | 1 | 1.06 | 4.39 | 0.98 | 2.84 |
| 37 | F | 74 | 2 | 25 | 1.00 | 4.33 | 1.91 | 2.55 |
| 38 | F | 63 | 15 | 2 | 1.17 | 3.73 | 1.25 | 1.85 |
| 39 | F | 66 | 4 | 0 | 1.32 | 3.31 | 0.76 | 2.00 |
| 40 | F | 52 | 7 | 7 | 0.96 | 3.39 | 0.99 | 1.98 |
| 41 | F | 42 | 11 | 1 | 1.44 | 3.89 | 1.79 | 2.01 |
| 42 | F | 49 | 0 | 0 | 1.05 | 3.70 | 1.16 | 2.07 |
| 43 | M | 70 | 9 | 22 | 0.96 | 3.73 | 1.10 | 2.15 |
| 44 | F | 54 | 26 | 12 | 0.97 | 3.15 | 1.02 | 1.85 |
| 45 | F | 56 | 20 | 18 | 1.08 | 4.66 | 0.79 | 2.97 |
| 46 | F | 55 | 26 | 2 | 1.45 | 5.37 | 1.55 | 3.16 |
| 47 | F | 66 | 24 | 2 | 1.59 | 4.75 | 1.19 | 2.84 |
| 48 | F | 42 | 16 | 0 | 1.42 | 4.54 | 1.73 | 2.42 |
| 49 | F | 42 | 26 | 12 | 0.91 | 3.62 | 1.02 | 2.39 |
| 50 | F | 58 | 24 | 1 | 1.93 | 5.18 | 1.49 | 2.34 |
| 51 | M | 63 | 19 | 2 | 0.95 | 4.67 | 1.67 | 4.37 |
| 52 | M | 47 | 8 | 22 | 1.18 | 4.48 | 1.64 | 2.44 |
| 53 | F | 42 | 1 | 1 | 2.60 | 6.11 | 1.33 | 3.74 |
| 54 | F | 30 | 5 | 24 | 0.56 | 2.96 | 1.30 | 1.37 |
| 55 | F | 39 | 28 | 28 | 0.67 | 4.17 | 2.72 | 1.40 |
| 56 | F | 54 | 2 | 0 | 1.29 | 4.20 | 1.18 | 2.61 |
| 57 | F | 67 | 11 | 21 | 0.79 | 3.13 | 1.18 | 1.67 |
| 58 | F | 58 | 15 | 9 | 0.77 | 4.20 | 1.48 | 2.39 |
| 59 | F | 63 | 5 | 23 | 1.53 | 6.16 | 2.29 | 3.41 |
| 60 | F | 45 | 14 | 5 | 0.92 | 3.59 | 1.44 | 1.94 |
| 61 | F | 50 | 3 | 3 | 0.59 | 2.82 | 1.12 | 1.58 |
| 62 | M | 42 | 10 | 10 | 0.89 | 4.68 | 1.04 | 2.96 |
| 63 | F | 45 | 14 | 5 | 0.92 | 3.59 | 1.44 | 1.94 |
| 64 | F | 52 | 13 | 1 | 0.77 | 5.70 | 1.73 | 3.62 |
| 65 | F | 63 | 26 | 23 | 0.94 | 3.70 | 1.23 | 1.84 |
| 66 | F | 73 | 26 | 22 | 1.16 | 4.44 | 1.55 | 2.29 |
| 67 | F | 42 | 8 | 6 | 1.30 | 3.94 | 0.92 | 2.55 |
| 68 | F | 38 | 18 | 12 | 1.06 | 3.68 | 1.09 | 2.16 |
| 69 | F | 59 | 26 | 26 | 1.50 | 6.25 | 1.38 | 3.80 |
| 70 | F | 69 | 24 | 24 | 0.89 | 3.04 | 0.87 | 1.63 |
| 71 | M | 70 | 12 | 12 | 1.10 | 3.48 | 1.14 | 1.58 |
| 72 | F | 43 | 6 | 3 | 0.95 | 4.84 | 1.45 | 3.23 |
| 73 | F | 39 | 28 | 4 | 1.44 | 4.35 | 1.17 | 2.76 |
| 74 | F | 37 | 1 | 1 | 0.83 | 3.64 | 1.52 | 1.78 |
| 75 | F | 64 | 16 | 12 | 0.91 | 4.19 | 1.54 | 2.51 |
| 76 | M | 73 | 0 | 0 | 0.67 | 3.21 | 1.00 | 1.81 |
| 77 | F | 68 | 26 | 20 | 4.82 | 6.52 | 1.24 | 3.84 |
| 78 | M | 62 | 20 | 20 | 3.15 | 5.42 | 0.92 | 3.13 |
| 79 | F | 58 | 26 | 2 | 2.74 | 4.18 | 1.37 | 2.09 |
| 80 | F | 34 | 16 | 16 | 1.62 | 6.01 | 1.64 | 3.64 |
| 81 | F | 43 | 8 | 8 | 1.17 | 4.36 | 1.35 | 2.46 |
| 82 | F | 43 | 28 | 28 | 0.55 | 3.15 | 0.97 | 1.96 |
| 83 | F | 46 | 24 | 5 | 1.05 | 3.34 | 0.84 | 1.99 |
| 84 | F | 75 | 11 | 7 | 1.15 | 4.78 | 1.26 | 2.94 |
| 85 | F | 43 | 28 | 28 | 0.55 | 3.15 | 0.97 | 1.96 |
| 86 | F | 46 | 12 | 12 | 1.96 | 4.04 | 0.83 | 2.60 |
| 87 | M | 42 | 6 | 2 | 1.41 | 5.17 | 1.18 | 3.25 |
| 88 | F | 29 | 26 | 26 | 0.98 | 2.25 | 1.05 | 0.84 |
| 89 | F | 38 | 5 | 5 | 2.71 | 0.94 | 0.94 | 1.29 |
| 90 | F | 69 | 10 | 4 | 0.99 | 3.35 | 1.10 | 1.73 |
| 91 | M | 68 | 16 | 10 | 0.77 | 4.29 | 1.29 | 2.58 |
| 92 | F | 38 | 17 | 1 | 1.40 | 3.22 | 1.03 | 1.68 |
| 93 | F | 68 | 26 | 26 | 0.83 | 4.47 | 1.38 | 2.67 |
| 94 | F | 41 | 26 | 4 | 0.33 | 4.32 | 1.97 | 2.33 |
| 95 | M | 54 | 22 | 22 | 1.95 | 5.19 | 1.43 | 3.32 |
| 96 | F | 76 | 10 | 10 | 0.94 | 6.70 | 2.59 | 3.64 |
| 97 | M | 44 | 2 | 2 | 2.73 | 4.29 | 1.09 | 1.72 |
| 98 | F | 57 | 26 | 26 | 1.33 | 3.25 | 0.93 | 1.73 |
| 99 | M | 33 | 16 | 2 | 0.54 | 3.88 | 1.41 | 2.09 |
| 100 | F | 50 | 28 | 28 | 1.49 | 4.23 | 0.99 | 2.76 |
| 101 | M | 59 | 26 | 22 | 1.06 | 3.28 | 1.01 | 1.63 |
| 102 | F | 28 | 22 | 22 | 0.94 | 4.38 | 1.35 | 2.83 |
| 103 | M | 68 | 28 | 6 | 1.50 | 4.62 | 1.07 | 2.74 |
| 104 | M | 57 | 6 | 2 | 0.69 | 3.98 | 1.69 | 2.10 |
| 105 | F | 58 | 12 | 12 | 1.42 | 4.56 | 1.26 | 2.72 |
| 106 | F | 50 | 28 | 28 | 1.49 | 4.23 | 0.99 | 2.76 |
| 107 | M | 62 | 28 | 28 | 0.80 | 3.23 | 1.31 | 1.50 |
| 108 | F | 63 | 12 | 12 | 0.84 | 5.31 | 2.14 | 2.96 |
| 109 | F | 29 | 26 | 26 | 0.98 | 2.25 | 1.05 | 0.84 |
| 110 | F | 58 | 12 | 12 | 1.33 | 5.06 | 1.53 | 3.13 |
| 111 | M | 33 | 16 | 2 | 0.54 | 3.88 | 1.41 | 2.09 |
| 112 | F | 71 | 2 | 2 | 1.85 | 4.99 | 1.43 | 3.32 |
| 113 | F | 41 | 12 | 9 | 1.03 | 3.39 | 0.94 | 2.12 |
| 114 | F | 41 | 26 | 4 | 0.33 | 4.32 | 1.97 | 2.33 |
| 115 | F | 58 | 18 | 2 | 0.83 | 3.82 | 2.00 | 1.45 |
| 116 | F | 44 | 24 | 22 | 1.26 | 4.41 | 1.62 | 2.39 |
| 117 | F | 68 | 2 | 2 | 1.30 | 4.18 | 1.19 | 2.47 |
| 118 | F | 71 | 10 | 1 | 1.92 | 5.07 | 1.08 | 2.62 |
| 119 | F | 52 | 14 | 9 | 0.82 | 3.78 | 1.70 | 1.96 |
| 120 | M | 58 | 22 | 4 | 0.91 | 2.36 | 0.64 | 1.14 |
| 121 | M | 62 | 26 | 10 | 1.61 | 4.94 | 1.80 | 2.79 |
| 122 | F | 55 | 15 | 15 | 0.74 | 3.99 | 1.16 | 2.41 |
| 123 | F | 52 | 22 | 22 | 1.60 | 4.46 | 1.40 | 2.45 |
| 124 | F | 58 | 12 | 12 | 1.03 | 4.37 | 1.91 | 2.31 |
| 125 | F | 38 | 24 | 14 | 1.74 | 3.57 | 0.99 | 1.92 |
| 126 | F | 64 | 26 | 26 | 1.32 | 3.77 | 1.33 | 1.84 |
| 127 | F | 51 | 26 | 23 | 1.54 | 4.97 | 1.16 | 3.10 |
| 128 | M | 60 | 20 | 20 | 1.70 | 5.88 | 2.34 | 2.86 |
| 129 | F | 69 | 18 | 18 | 1.14 | 4.86 | 1.99 | 2.51 |
| 130 | F | 47 | 4 | 4 | 1.72 | 5.06 | 1.94 | 2.63 |
| 131 | F | 62 | 20 | 20 | 0.92 | 4.84 | 1.95 | 2.18 |
| 132 | F | 62 | 28 | 24 | 1.55 | 3.32 | 0.76 | 1.92 |
| 133 | M | 46 | 8 | 4 | 1.30 | 3.93 | 0.82 | 2.49 |
| 134 | M | 61 | 28 | 28 | 0.76 | 4.73 | 1.35 | 2.85 |
| 135 | F | 60 | 6 | 0 | 0.89 | 4.96 | 1.83 | 2.86 |
| 136 | M | 60 | 8 | 4 | 1.26 | 7.02 | 1.94 | 4.33 |
| 137 | F | 64 | 22 | 22 | 0.59 | 1.93 | 0.62 | 1.01 |
| 138 | F | 49 | 24 | 24 | 1.16 | 4.36 | 1.24 | 2.56 |
| 139 | M | 62 | 24 | 24 | 2.07 | 4.44 | 1.12 | 2.70 |
| 140 | M | 66 | 12 | 10 | 0.73 | 4.89 | 1.27 | 3.10 |
| 141 | F | 41 | 28 | 12 | 0.72 | 3.08 | 1.44 | 1.42 |
| 142 | F | 62 | 18 | 4 | 1.89 | 3.53 | 1.28 | 1.56 |
| 143 | M | 64 | 28 | 10 | 1.96 | 5.28 | 1.62 | 3.13 |
| 144 | F | 46 | 14 | 5 | 0.92 | 3.59 | 1.44 | 1.94 |
| 145 | F | 65 | 28 | 28 | 1.67 | 3.65 | 1.20 | 1.77 |
| 146 | F | 62 | 28 | 28 | 1.49 | 4.01 | 1.13 | 2.17 |
| 147 | M | 40 | 28 | 28 | 1.29 | 3.68 | 0.82 | 2.28 |
| 148 | F | 69 | 1 | 1 | 0.92 | 3.91 | 1.40 | 2.54 |
| 149 | F | 43 | 6 | 3 | 0.95 | 4.84 | 1.45 | 3.23 |
| 150 | F | 67 | 28 | 20 | 2.05 | 5.39 | 1.21 | 3.25 |
| 151 | F | 49 | 8 | 4 | 0.66 | 4.00 | 1.79 | 2.08 |
| 152 | F | 72 | 26 | 1 | 0.96 | 3.55 | 1.16 | 1.88 |
| 153 | F | 75 | 28 | 28 | 1.28 | 4.14 | 1.18 | 2.37 |
| 154 | M | 27 | 3 | 1 | 0.42 | 2.58 | 1.24 | 1.15 |
| 155 | F | 72 | 15 | 0 | 1.07 | 3.79 | 1.14 | 2.08 |
| 156 | M | 45 | 22 | 22 | 1.83 | 5.78 | 0.91 | 4.22 |
| 157 | F | 24 | 28 | 22 | 1.72 | 3.04 | 0.79 | 1.56 |
| 158 | F | 62 | 28 | 22 | 0.74 | 2.61 | 1.01 | 1.16 |
